# Characterization of ACE Inhibitory Peptides Prepared from *Pyropia pseudolinearis* Protein

**DOI:** 10.3390/md19040200

**Published:** 2021-04-01

**Authors:** Yuya Kumagai, Keigo Toji, Satoshi Katsukura, Rie Morikawa, Toshiki Uji, Hajime Yasui, Takeshi Shimizu, Hideki Kishimura

**Affiliations:** 1 Laboratory of Marine Chemical Resource Development, Faculty of Fisheries Sciences, Hokkaido University, Hakodate, Hokkaido 041-8611, Japan; yuyakumagai@fish.hokudai.ac.jp; 2 Chair of Marine Chemical Resource Development, Graduate School of Fisheries Sciences, Hokkaido University, Hakodate, Hokkaido 041-8611, Japan; Keigo_Toji@kirin.co.jp (K.T.); satoshi.katsukura@meiji.com (S.K.); k3996959@eis.hokudai.ac.jp (R.M.); 3 Laboratory of Aquaculture Genetics and Genomics, Faculty of Fisheries Sciences, Hokkaido University, Hakodate, Hokkaido 041-8611, Japan; t-uji@fish.hokudai.ac.jp; 4 Laboratory of Humans and the Ocean, Faculty of Fisheries Sciences, Hokkaido University, Hakodate, Hokkaido 041-8611, Japan; hagime@fish.hokudai.ac.jp; 5 Hokkaido Industrial Technology Center, Department of Research and Development, Hakodate, Hokkaido 041-0801, Japan; shimizu@techakodate.or.jp

**Keywords:** red alga, *Pyropia pseudolinearis*, Uppurui Nori, ACE inhibitory peptides, docking simulation

## Abstract

More than 7000 red algae species have been classified. Although most of them are underused, they are a protein-rich marine resource. The hydrolysates of red algal proteins are good candidates for the inhibition of the angiotensin-I-converting enzyme (ACE). The ACE is one of the key factors for cardiovascular disease, and the inhibition of ACE activity is related to the prevention of high blood pressure. To better understand the relationship between the hydrolysates of red algal proteins and the inhibition of ACE activity, we attempted to identify novel ACE inhibitory peptides from *Pyropia pseudolinearis*. We prepared water soluble proteins (WSP) containing phycoerythrin, phycocyanin, allophycocyanin, and ribulose 1,5-bisphosphate carboxylase/oxygenase. In vitro analysis showed that the thermolysin hydrolysate of the WSP had high ACE inhibitory activity compared to that of WSP. We then identified 42 peptides in the hydrolysate by high-performance liquid chromatography and mass spectrometry. Among 42 peptides, 23 peptides were found in chloroplast proteins. We then synthesized the uncharacterized peptides ARY, YLR, and LRM and measured the ACE inhibitory activity. LRM showed a low IC_50_ value (0.15 μmol) compared to ARY and YLR (1.3 and 5.8 μmol). In silico analysis revealed that the LRM sequence was conserved in cpcA from Bangiales and Florideophyceae, indicating that the novel ACE inhibitory peptide LRM was highly conserved in red algae.

## 1. Introduction

Japan’s Ministry of Agriculture, Forestry and Fisheries promotes “*Shokuiku*” (Food and Nutrition Education). The guideline recommends eating well-balanced meals combined with vegetables, fruits, milk products, beans, and fish. In addition, information of the nutritional and bioactive functions of foods based on scientific evidence is required. The importance of such food-derived components has been recognized not only in Japan but also worldwide. One of the examples of functional components is bioactive peptide. Until now, there have been various reports for the functions of bioactive peptides, such as decreases in blood pressure, glucose, cholesterol, and neutral lipids and increases in immune response [1]. Among these peptides, several antihypertensive peptides from natural sources, such as sesame, milk, sardine, and seaweed have been used as food for specified health uses (FOSHU) in Japan [2,3].

Since high blood pressure (hypertension) has no obvious indicating symptoms, it is called a “Silent Killer”. Hypertension is a major risk for cardiovascular diseases and causes serious health problems regarding heart failure, atrial fibrillation, chronic kidney disease, heart valve diseases, aortic syndromes, and dementia [4,5]. The renin-angiotensin system (RAS) is one of the blood pressure regulators. The angiotensin-I-converting enzyme (ACE: EC 3.4.15.1), which is a dipeptidyl carboxypeptidase, is a key enzyme in RAS. The enzyme converts the inactive angiotensin I to an active angiotensin II, resulting in an increase in blood pressure [6]. Several ACE inhibitory drugs including captopril, enalapril, lisinopril, and alacepril are used, but these drugs are normally associated with side effects such as dry cough, angioedema, taste disturbance, and skin rash [4,6,7]. Hence, ACE inhibitory peptides derived from natural sources are desired for the preventive therapy of high blood pressure.

On the other hand, Japanese people have been using seaweed as a food source and industrial material since ancient times. Recently, the health functions of seaweed’s nutritional components have been reported [8,9,10,11,12,13,14,15,16,17,18,19,20]. In particular, the idea of using seaweed proteins as bioactive peptides has attracted much attention [21]. Among seaweeds, the protein content of red algae tends to be higher than that of brown and green algae [22]. Red algae are currently classified into seven classes—Bangiophyceae, Compsopogonophyceae, Cyanidiophyceae, Florideophyceae, Porphyridiophyceae, Rhodellophyceae, and Stylonematophyceae [23], which are composed of more than 7000 species in the world [24] and 800 species on the coast of Japan. However, only a limited number of species such as nori (*Neopyropia* sp.) and agar (*Gelidium* sp. and *Gracilaria* sp.) are used as industrial foodstuffs, and some species such as *Grateloupia asiatica*, *Gloiopeltis furcata*, *Chondria crassicaulis*, and *Nemalion vermiculare* are consumed as local food. Therefore, other red algae are also expected to be used effectively as food material and a source of bioactive components. The main proteins in red algae are phycobiliproteins that form phycobilisome in a chloroplast and play a light-harvesting role in photosynthesis [25]. Phycobiliproteins primarily consist of phycoerythrin (PE), phycocyanin (PC), and allophycocyanin (APC), and these proteins commonly possess α- and β-subunits [26,27]. APC constitutes the core domain of phycobilisomes, while PE and PC are components of the rods. Since PE is at the tip region of the rods, it is easily released in water layers accompanied by ribulose 1,5-bisphosphate carboxylase/oxygenase (Rubisco) [28]. Regarding the peptides in the hydrolysates of phycobiliproteins and of Rubisco from red algae, many reports on their health benefit have been published, i.e., their ACE inhibitory activity [29,30,31,32,33,34,35,36], renin inhibitory activity [37], DPP-IV inhibitory activity [38], antioxidant activity [33,39,40,41], anti-inflammatory activity [42], and antiplatelet aggregation activity [43].

Since red algal phycobiliproteins are commonly composed of many hydrophobic and aromatic amino acid residues (>50%), thermolysin is suitable for the preparation of ACE inhibitory peptides [26,35,44,45]. In our previous study, we attempted to understand the correlation between in vitro and in silico analyses using IC_50_ values of ACE inhibitory peptides prepared by the thermolysin hydrolysis of phycobiliproteins and Rubisco [46,47]. The in vitro ACE inhibitory activity was well correlated with the IC_50_ value from in silico analysis. Namely, the in silico approach has the potential to predict the biological activity of peptides derived from red algal protein. However, there were slight gaps in the correlation due to the lack of information on ACE inhibitory peptides from red algae. This indicates that the hydrolysates of red algal phycobiliproteins and Rubisco still contain uncovered ACE inhibitory peptides. Therefore, we used *Pyropia pseudolinearis*, which is called “Uppurui Nori” in Japan and is eaten in certain local areas, as a sample in this study. We first extracted phycobiliproteins and Rubisco as water-soluble proteins (WSP) and prepared their hydrolysates. We then identified the ACE inhibitory peptides in the hydrolysate and found three novel peptides. Further, we determined IC_50_ values using synthetic peptides and performed docking simulation between the ACE and three peptides. We also determined the nucleotide sequence of the chloroplast proteins of PE, PC, and APC to reveal the source of the peptides. This study is useful for the evaluation of proteins from red algae as a source of ACE inhibitory peptides.

## 2. Results and Discussion

### 2.1. Properties and ACE Inhibitory Activities of Water Soluble Protein (WSP) and the Thermolysin Hydrolysate of the WSP

**Figure 1:**
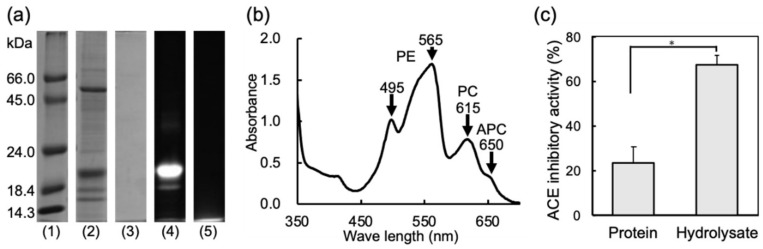
Properties and ACE inhibitory activities of *P. pseudolinearis* WSP and its hydrolysate. (**a**) SDS-PAGE. Lane 1, Maker; Lane 2, WSP; Lane 3, the thermolysin hydrolysate of the WSP; Lane 4, WSP (Fluorescence); Lane 5, the thermolysin hydrolysate of the WSP (Fluorescence). (**b**) Visual ray absorption spectra of WSP. (**c**) ACE inhibitory activities of WSP and the hydrolysate. Bars represent standard errors. * *p* < 0.05.

To evaluate the potential of ACE inhibitory activity from *P. pseudolinearis*, we first prepared WSP and its thermolysin hydrolysate. The composition of WSP was confirmed by SDS-PAGE and spectral analysis. The components of WSP were mainly found to be 20,000 and 55,000 MW (Figure 1a). The former band was fluoresced by the irradiation of excitation light (490–560 nm), suggesting phycobiliproteins. The maximum absorption peaks of WSP were found at around 495, 565, 615, and 650 nm (Figure 1b). These peaks corresponded to PE (495 and 565 nm), PC (615 nm), and APC (650 nm). The absorbance of PE was high compared with PC and APC. This result indicated that the main component of 20,000 MW was PE. A previous study revealed that 55,000 MW of the protein in the water-extract of red algae was the Rubisco large subunit [47], suggesting that the WSP of *P. pseudolinearis* also contained PE and the Rubisco large subunit as main components. The digestion of proteins was confirmed by SDS-PAGE, showing that protein bands were completely hydrolyzed (Figure 1a). The hydrolysate of the WSP was centrifuged at 4 °C, 15,000× *g*, for 10 min, and the supernatant was lyophilized and used for the detection of ACE inhibitory peptides. We compared the ACE inhibitory activities of WSP and the hydrolysate (Figure 1c). WSP inhibited 23.6% of the ACE activity, while the hydrolysate of the WSP inhibited 67.7% activity, showing that the hydrolysate contained ACE inhibitory peptides. It is thought that the ACE inhibitory activity of the peptide is affected by its amino acid sequence and molecular weight. In the present study, to evaluate proteins from *P. pseudolinearis* as a source of ACE inhibitory peptides, we prepared its hydrolysate in the same method used in our previous study [35]. We identified 15 tripeptides, 10 tetrapeptides, 11 pentapeptides, and 6 hexapeptides, and these peptides’ compositions were similar to that of our previous result [35]. In the future, we would like to study the optimal condition under which peptides are prepared with the highest inhibitory activity by adjusting the hydrolysis time. We afterwards attempted to identify ACE inhibitory peptides by HPLC.

### 2.2. Fractionation of the Hydrolysate of the WSP

**Figure 2:**
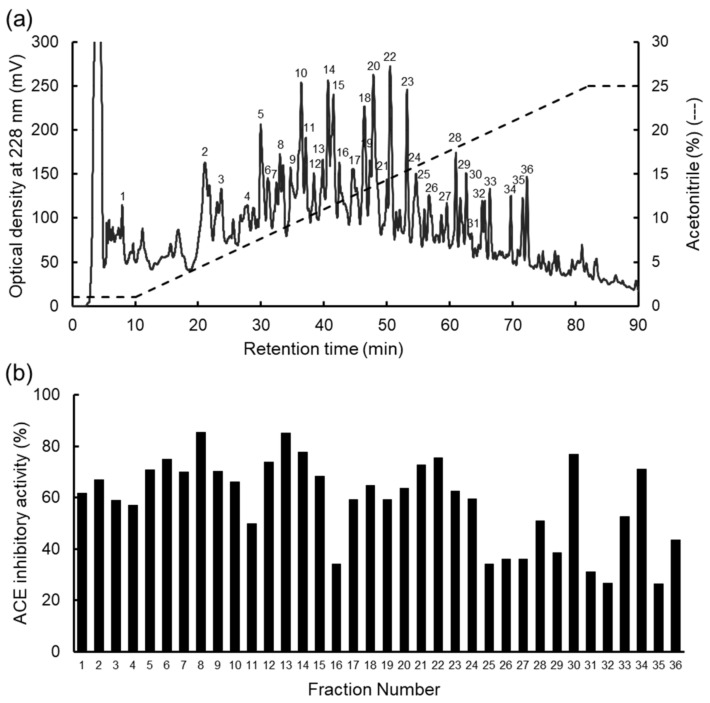
HPLC chromatogram of the WSP hydrolysate and ACE inhibitory activity. (**a**) Chromatogram of the WSP hydrolysate by HPLC. Peptides were separated by Mightysil RP-18GP column. The numbers on the peaks (1–36) were pooled. (**b**) ACE inhibitory activity of each fraction (1–36).

To determine the ACE inhibitory peptides, fractionation was performed by RP-HPLC (Figure 2a). Thirty-six fractions were obtained by HPLC. The ACE inhibitory activity was measured in each fraction (Figure 2b), so the activity was detected in all fractions. We have previously prepared the thermolysin hydrolysates of WSP from dulse [35], *Mazzaella japonica* [26,36], and *G. asiatica* [47], which showed that the patterns of the chromatogram differed, even though the main WSP components of these red algae were phycobiliproteins and Rubisco. The hydrolysate of the WSP from *P. pseudolinearis* contained many peptides. Peptide peaks, which were eluted after 22% acetonitrile (FN 34–36), were not found in the other hydrolysates. The peaks of hydrolysates from dulse and *G. asiatica* were detected with up to 15% acetonitrile, and the peak from *M. japonica* was detected with up to 18% acetonitrile. The difference might be due to minor proteins detected on SDS-PAGE. Therefore, we attempted to determine the peptide structures in all fractions.

### 2.3. Identification of Peptides from the WSP

Since we detected the ACE inhibitory activity in 36 fractions, peptide sequences were determined by MALDI-TOF/MS/MS. Among 36 fractions, 42 peptides were determined (Table 1). The complete chloroplast genome of *Pyropia pulchra* was determined [48,49], which is the related species in *P. pseudolinearis* [50]. We employed the *P. pulchra* chloroplast genome and performed in silico thermolysin digestion in it (Table 1). Among the 42 peptides, 23 kinds of peptides were found in chloroplast proteins, which were constituted of 101 peptide sequences from 66 chloroplast proteins. Among them, it was found that 31 peptides were obtained by in silico digestion. Although the peptides VRFK, FFR, FAR, LRM, FRV, and FGRPF in Table 1 were degraded by in silico thermolysin digestion, the peptides were detected in in vitro digestion. Peptide LDY, which was identified as an ACE inhibitory peptide, was coded on 17 chloroplast proteins, including phycobiliproteins apcA, apcB, cpcA, and cpeA. Peptide YLR, which was coded on 12 chloroplast proteins, was not identified as an ACE inhibitory peptide and produced by in silico digestion. Peptide LRY, which was identified as an ACE inhibitory peptide, was coded on 8 chloroplast proteins, including apcB, cpcB, and cpeB, and produced by in silico digestion. Contradictions, i.e., where some peptides were detected in vitro but not produced by in silico analysis, were due to an insufficient understanding of the protease reaction in the complex protein sources. To confirm the sequences in Uppurui Nori, we then determined the nucleotide sequences of phycobiliproteins from *P. pseudolinearis*.

### 2.4. Sequencing and Comparison of Phycobiliproteins

We employed *P. pulchra* chloroplast proteins to find the protein sources of the produced peptides. Phycobiliproteins are the major proteins in red algae. Therefore, we determined the protein sequencing of the phycobiliproteins. Phycobiliproteins (PE, PC, and ACP) were composed of α- and β-chains, and the gene coding regions of these chains were continually coded with a short AT-rich spacer. The sequences were deposited in the DNA Data Bank of Japan (DDBJ) as LC599086-LC599088. The phycobiliprotein sequences of *P. pseudolinearis* were compared to those of other red algae. High identities were obtained in the Bangiophyceae species (>98.8%) and in Florideophyceae species (>88.1%) (Table 2). We employed two sequences from Florideophyceae (*Palmaria palmata* in Japan and *G. asiatica*), which were thought to be a related species (Nemaliophycidae) and a distant species (Rhodymeniophycidae) [51]. The lowest identity of the sequence was obtained from the cpeB of the distant species *P. palmata* in Japan, indicating that Florideophyceae identities were constant. The data in Table 1 were obtained from *P. pulchra*, and the differences in phycobiliproteins between *P. pseudolinearis* and *P. pulchra* were cpcB and apcA. The differences were not involved in the ACE inhibitory peptide sequences, indicating that ACE inhibitory peptides from phycobiliproteins were also conserved in *P. pseudolinearis.*

### 2.5. Identification of Novel ACE Inhibitory Peptides from Red Algae

Among the predicted 42 peptides, we focused on the peptides from phycobiliproteins since phycobiliproteins were the major components of the WSP. We previously determined several ACE inhibitory peptides from Japanese dulse and presumed that the peptides LDY, LRY, VYRT, AGGEY, VDHY, LKNPG, and YRD were from phycobiliproteins [35]. To find novel ACE inhibitory peptides, we focused on the rest of the peptides from phycobiliproteins. Among 42 peptides, 9 were from phycobiliproteins. Three were already identified as ACE inhibitory peptides using synthetic peptides (Table 3). Wu et al. reported that the tripeptides having an amino acid sequence of aromatic, basic, and hydrophobic in order from the N-terminus possessed high ACE inhibition activity [52]. Following the tripeptide rule in ACE inhibition, three candidate peptides were selected: ARY, YLR and LRM. We then synthesized the three peptides and measured the activity. ARY and YLR, which follows one tripeptide rule (ARY in arginine and YLR in tyrosine), showed 1.3 and 5.8 μmol IC_50_ values, respectively (Table 3). LRM, which follows two tripeptide rules (LRM in arginine and methionine), showed a low IC_50_ value (0.15 μmol) compared to ARY and YLR. LRM in cpcA and YLR in cpcA, apcA, and apcB were conserved in Bangiales and Florideophyceae. From these results, we identified three novel ACE inhibitory peptides (ARY, YLR, and LRM), which were highly conserved in red algae. Several ACE inhibitory peptides derived from nori (AKYSY) and sardines (VY) have been used as functional ingredients in Japan (FOSHU). In the present study, we found that LRM possessed relatively high ACE inhibitory activity. In the future, we would like to investigate the digestibility and absorption of ACE inhibitory peptides derived from red algae.

### 2.6. In Silico Analysis of Binding of Peptides and ACE

**Figure 3:**
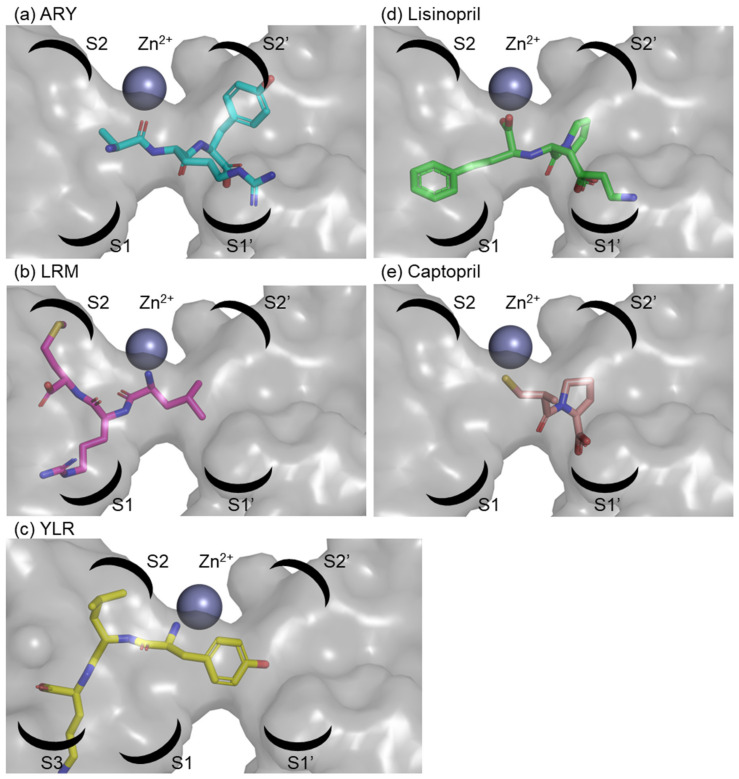
Binding motifs of ARY, LRM, and YLR in the active site of ACE. (**a**) ARY and ACE interaction; (**b**) LRM and ACE interaction; (**c**) YLR and ACE interaction; (**d**) Lisinopril and ACE interaction (PDB 1O86); (**e**) Captopril and ACE interaction (PDB 1UZF). Zinc ion is shown as a dark gray sphere. The binding pockets of the enzymes are labeled within the surface-rendered catalytic channel of the C-domain of ACE.

**Figure 4:**
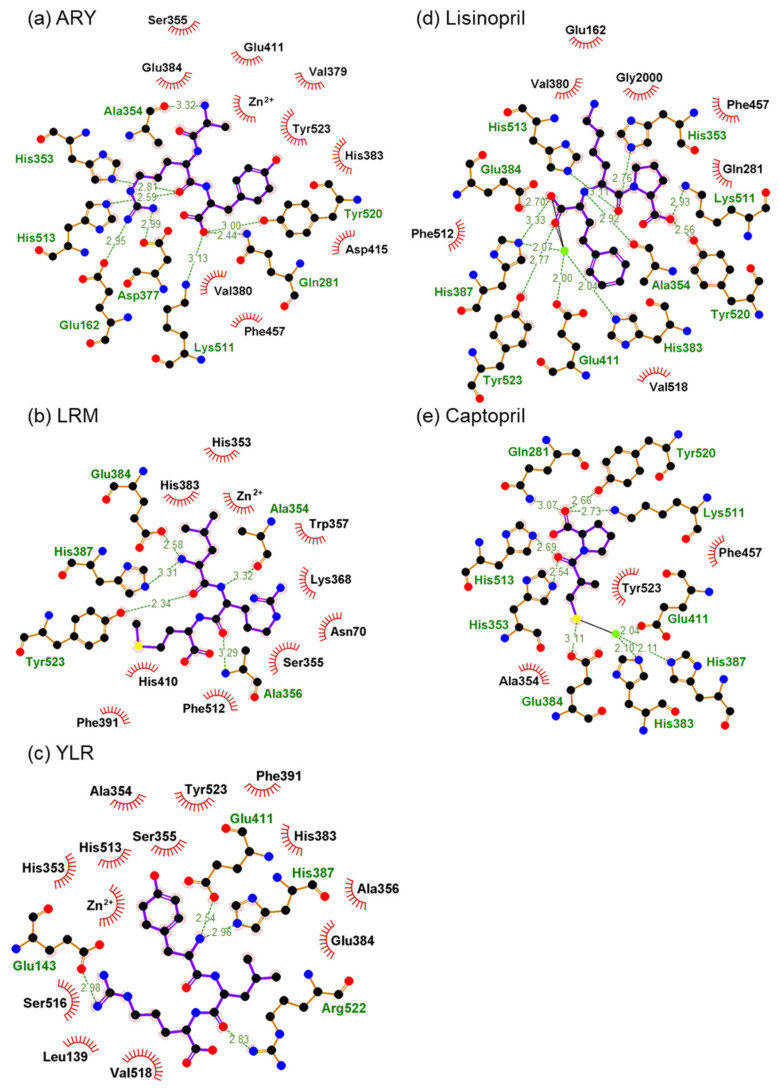
Two-dimensional molecular docking results for ARY, LRM, and YLR with ACE (PDB: 1O86). (**a**) ARY and ACE interaction; (**b**) LRM and ACE interaction; (**c**) YLR and ACE interaction; (**d**) Lisinopril and ACE interaction (PDB 1O86); (**e**) Captopril and ACE interaction (PDB 1UZF).

We identified three novel ACE inhibitory peptides (ARY, YLR, and LRM). Among them, LRM showed strong ACE inhibitory activity compared to the other two peptides. To know the inhibitory mechanism, the docking simulation between ACE and three peptides was performed by using the HPEPDOCK server. The docking energy scores with ACE were as follows: ARY: –155.685 kcal/mol; YLR: –166.290 kcal/mol; LRM: –140.683 kcal/mol. The results contradicted the in vitro synthetic peptide analysis. The binding of the three peptides in ACE was as follows: ARY was coordinated in the S1′ and S2′ pockets of the ACE, LRM was in the S2 and S1 pockets, and YLR was in the S3 and S2 pockets (Figure 3). Although they were coordinated near a zinc ion, their binding and zinc ions were not found. On the other hand, the ACE inhibitor Lisinopril was coordinated in the S1, S1′, and S2′ pockets (PDB, 1O86) containing the interaction with the zinc ion. The ACE inhibitor Captopril was coordinated in the S1′ and S2′ pockets (PDB, 1UZF) containing the interaction with the zinc ion. Bindings with peptides or inhibitors are shown as 2-D image in Figure 4. The edges of the two inhibitors were bound to the ACE. Namely, one side of Lisinopril was bound to the zinc ion, Glu384, and His387, and the other side was bound to Lys511 and Tyr520. This binding was also found in Captopril. One side of Captopril was bound to the zinc ion and Glu384, and the other side was bound to Gln281, Lys511, and Tyr520. Among the three peptides, one side of the main chain of YLR was bound to His387 and Glu411, one side of the main chain of LRM was bound to Glu384 and His387, and one side of the main chain of ARY was bound to Gln281, Lys511, and Tyr520. Although LRM showed strong ACE inhibition by in vitro synthetic peptide analysis, the inhibition was weak compared to that of the inhibitors (the IC_50_ value of Captopril was approximately 17–21 nM [53,54]). The difference might be due to the loss of interaction with the zinc ion.

## 3. Materials and Methods

### 3.1. Materials

Uppurui Nori, *P. pseudolinearis*, was collected from the coast of Hakodate, Japan. The sample was stored at –30 °C until use. ACE from rabbit lung was purchased from Sigma-Aldrich Co. (St. Louis, MO, USA). Millex-GV (pore size: 0.22 µm) and Millex-LG (pore size: 0.20 µm) were purchased from MERCK MILLIPORE Ltd. (Billerica, MA, USA). Synthetic peptides (purity: ≥95%) were purchased from Medical Biological Laboratories Co. (Nagoya, Japan). Hyppuryl-L-histidyl-L-leucine (Hip-His-Leu), thermolysin (EC 3.4.24.27) from *Bacillus thermoproteolyticus*, trifluoroacetic acid (TFA), and all other regents were purchased from Fujifilm Wako Pure Chemical (Osaka, Japan).

### 3.2. Preparation of the WSP and Its Hydrolysate

Preparation of the WSP and the hydrolysate from *P. pseudolinearis* was as per our previous studies [35,47]. Namely, the frozen *P. pseudolinearis* samples were lyophilized and ground into a fine powder by Wonder Blender WB-1 (OSAKA CHEMICAL Co., Osaka, Japan). The powder was dissolved in 20 v/w distilled water, and protein extraction was performed at 4 °C for 12 h. The WSP was recovered by centrifugation at 4 °C, 15,000× *g*, for 10 min, and the supernatant was then used as the WSP. The protein hydrolysate of the WSP was prepared by the digestion of protein extract with 1.0 wt. % thermolysin at 70 °C for 3 h. The reaction was stopped by boiling for 10 min. The visible ray absorption spectrum of the WSP was analyzed by a spectrophotometer (UV-1800, SHIMADZU, Kyoto, Japan). Sodium dodecyl sulfate-polyacrylamide gel electrophoresis (SDS-PAGE) was performed by Laemmli’s method with a 0.1% SDS-13.75% polyacrylamide slab gel [55]. The gel was stained with 0.1% Coomassie Brilliant Blue (CBB) R-250 in 50% methanol–7% acetic acid, and the background of the gel was decolorized with 7% acetic acid. Fluorescence of phycobiliproteins on the slab gel was detected by gel documentation LED illuminator (VISIRAYS AE-6935GN: ATTO, Tokyo, Japan).

### 3.3. Separation of WSP Hydrolysate and Determination of Peptide Structures

The hydrolysate of the WSP was separated by RP-HPLC. The hydrolysate was dissolved (20 mg/mL) in ultra-pure water containing 0.1% TFA and applied to sequential filtration by Millex-GV and Millex-LG. An aliquot of the sample (100 μL) was applied to a Mightysil RP-18GP column (4.6 × 150 mm) (Kanto Kagaku, Tokyo, Japan) and eluted with a linear gradient of acetonitrile from 1–25% containing 0.1% TFA at flow rate of 1.0 mL/min. Absorbance of the eluent was monitored at 228 nm. The eluted peptide fractions were collected and labeled 1–36. The fractions were used for the ACE inhibitory assay. The amino acid sequences of peptides were determined by Matrix Assisted Laser Desorption / Ionization Time of Flight Tandem Mass Spectrometry (MALDI-TOF/MS/MS) using a 4700 Proteomics Analyzer with DeNovo Explorer software version 3.6 (Applied Biosystems, Carlsbad, CA, USA). α-Cyano-4-hydroxycinnamic acid was used as matrix.

### 3.4. Assay of ACE Inhibitory Activity

The assay was performed using the same method as described previously [35]. The ACE inhibitory activities of the WSP, WSP hydrolysate, and synthetic peptides were measured in triplicate, and the mean ± standard error for each was calculated. Statistical analyses were carried out using the Student’s *t* test. In this study, the IC_50_ was defined as an absolute quantity of the peptide to inhibit 50% of 1.0 U ACE.

### 3.5. Extraction and Sequencing of Chloroplast DNA

Chloroplast DNA was extracted from the thalli of *P. pseudolinearis* according to the CTAB method with some modifications [56,57,58]. The nucleotide sequences of chloroplast DNA were analyzed by using a next generation sequencer, the Ion PGM System (Thermo Fisher SCIENTIFIC, Waltham, MA, USA). The data were assembled with CLC Genomics Workbench 9.5.4 (QIAGEN, Hilden, Germany). Nucleotide and deduced amino acid sequences of *P. pseudolinearis* phycobiliproteins were detected using BLAST (Basic Local Alignment Search Tool). The sequences of phycobiliproteins were deposited as DDBJ Accession No. LC599086-LC599088.

### 3.6. Bioinformatics Analysis of ACE Inhibitory Peptides

The information on the reported ACE inhibitory peptide was obtained from the BIOPEP-UWM database (http://www.uwm.edu.pl/biochemia/index.php/pl/biopep) on 11 November 2020. From the database, 1019 ACE inhibitory peptides were extracted. The peptide sequences from Uppurui Nori were manually annotated. The thermolysin-digestion products of chloroplast proteins were predicted using PeptideCutter (https://web.expasy.org/peptide_cutter/). Docking simulation of the ACE inhibitory peptide with the ACE was performed using the HPEPDOCK server [59] using default parameters. The ACE from human testis complex with lisinopril (PDB code 1O86) was employed for docking simulation. Before docking, the inhibitor and water molecules were removed, and hydrogens were added.

## 4. Conclusions

In this study, we prepared a water-soluble protein (WSP) hydrolysate from the red alga *P. pseudolinearis*, which mainly contained PE, PC, APC, and Rubisco. We then determined the ACE inhibitory activity in the thermolysin hydrolysate of the WSP. We identified 42 peptides by HPLC and MALDI-TOF/MS/MS and confirmed that 23 peptides were from chloroplast proteins. The synthetic peptide analyses showed that ARY, YLR, and LRM were the ACE inhibitory peptides. Among them, LRM showed a low IC_50_ value (0.15 μM) compared with ARY and YLR (1.3 and 5.8 μM). Docking simulation analyses showed that three peptides were coordinated in the active site of the ACE. However, these peptides do not bind to the zinc ion, which may be the reason for their relatively low ACE inhibitory activity. Among these three peptides, LRM was conserved in the primary structure of cpcA from Bangiales and Florideophyceae, indicating that the novel ACE inhibitory peptide LRM can be obtained from many red algae. Therefore, red algae have the potential not only as a protein source but also as an ingredient for supplements and functional foods for human. In a following study, we will seek to investigate how effective any practical applications of the WSP hydrolysate are.

## Figures and Tables

**Table 1:** ACE inhibitory peptide sequences in the WSP from *P. pseudolinearis* and identification of the peptide sources from in silico digestion of *P. pulchra* plastid proteins.

FN	Peptide	Number of Peptides in Chloroplast Proteins	In Silico Thermolysin Digestion	
			◯ ^a^	× ^b^
3	AYR	6	rbcL	apcE, atpA, cpcG, psaL, rps7
4	MTFF	0	- ^c^	-
5	VRFK ^d^	1	gltB	-
6	KFR	4	-	accA, cemA, chlI, rpl16
6	WHKPA	0	-	-
6	FFKWEF	0	-	-
7	FGGR	1	petJ	-
7	LVER	0	-	-
8	YRD	7	-	cpeA, moeB, odpA, orf148, rps14, ycf3, ycf21
8	FFR ^d^	4	psaB, ycf22	psbT, ycf63
9	ARY	6	atpI, chlB, gltB	apcA, orf263, ycf46
9	RFR	3	-	odpA, rpoC1 (2) ^e^
10	FAR ^d^	7	clpC, orf174, ycf26	atpI, chlB, cpeA, ilvH
10	YLR	12	-	accA, apcA, apcB, apcD, apcE, apcF, carA, cpcA, orf114, rpl3, rpl19, rps9
10	VYRT	1	-	cpeA
10	FVCG	0	-	-
11	FFLREF	0	-	-
12	SRGL	1	-	rpl23
13	ACWR	0	-	-
13	RFAPR	0	-	-
14	ACPSGT	0	-	-
16	WER	1	-	psbA
17	LDY	17	chlN, cpcA, cpeA, infB, rbcL, ycf16	accA, apcA, apcB, atpA, infC, orf111, orf565, rpoA, rps1, syh, ycf24
17	LLEER	0	-	-
18	PGCRR	0	-	-
18	FLWWLR	0	-	-
20	AAGRFP	1	-	cpeA
23	LVFFGH	0	-	-
24	PVAFN	0	-	-
25	LRM ^d^	2	cpcA	psbB
26	LRY	8	apcB, apcE, apcF, cpcB, cpeB	apcD, apcE, rbcR
27	CPSNN	0	-	-
27	AWRRP	0	-	-
27	LWT	4	trpG	psaB, psbD, psbZ
28	YRF	3	-	cpcG, odpA, syh
28	FRV ^d^	8	petB, rbcL, rps5, rpoB (2), ycf26	pgmA, rpoB
29	VNLF	1	-	ycf38
29	PGDTY	0	-	-
30	EWYPH	0	-	-
32	KTFPY	0	-	-
34	FGRPF ^d^	1	rbcL	-
36	VESR	2	-	rbcL, rpoA
Total		101 peptides in 66 kinds of proteins (31 peptides were produced by in silico digestion)		

Data from *Pyropia pulchra* plastid (NC_029861.1). ^a^ The peptide is produced by thermolysin digestion from the protein. ^b^ The peptide is confirmed in the protein sequence, but it is not produced by thermolysin digestion. ^c^ “-” not detected in the plastid genome. ^d^ The peptides are hydrolyzed by in silico thermolysin digestion. Hydrolysis position of peptides show “-”: VR-FK; LR-M; FR-V; FGRP-F; F-FR; F-AR. ^e^ The parentheses indicate number of in silico thermolysin digestion site in proteins.

**Table 2:** Identity of phycobiliproteins from *P. pseudolinearis* with other red algae.

Species	Accession No.	Identity (%)					
		cpeA ^a^	cpeB ^a^	cpcA ^b^	cpcB ^b^	apcA ^c^	apcB ^c^
*Pyropia pulchra*	NC_029861.1	100	100	100	98.8	99.4	100
*Porphyra purpurea*	NC_000925	100	99.4	99.4	99.4	100	100
*Neopyropia yezoensis*	KC51072	100	100	100	98.3	100	100
*Palmaria palmata* in Japan	AB807662	89.0	88.1	88.9	90.1	95.0	98.1
*Grateloupia asiatica*	AP018129	90.2	91.5	92.6	90.1	96.3	93.8

^a^, DDBJ accession No. LC599086. ^b^, DDBJ accession No. LC599087. ^c^, DDBJ accession No. LC599088.

**Table 3:** ACE inhibitory activity of the synthetic peptide.

Peptide	IC_50_ (μmol)	Reference
ARY	1.3	This study
YLR	5.8	
LRM	0.15	
VYRT	0.14	[35]
LDY	6.1	
FEQWAS	>2.8	
LRY	0.044	

## Data Availability

Not applicable.
